# Youth Voucher Program in Madagascar Increases Access to Voluntary Family Planning and STI Services for Young People

**DOI:** 10.9745/GHSP-D-16-00321

**Published:** 2017-02-23

**Authors:** Eva Burke, Judy Gold, Lalaina Razafinirinasoa, Anna Mackay

**Affiliations:** a Independent consultant, London, England.; b Independent consultant, Melbourne, Australia.; c Marie Stopes Madagascar, Antananarivo, Madagascar.; d Marie Stopes International, New York, United States.

## Abstract

**Background:**

Young people often express a preference for seeking family planning information and services from the private sector. However, in many Marie Stopes International (MSI) social franchise networks, the proportion of young clients, and particularly those under 20 years of age, remains low. Marie Stopes Madagascar (MSM) piloted a youth voucher program that joins a supply-side intervention—youth-friendly social franchisee training and quality monitoring—with a corresponding demand-side-component, free vouchers that reduce financial barriers to family planning access for young people.

**Methods:**

Young people identified by MSM's community health educators (CHEs) received a free voucher redeemable at a BlueStar social franchisee for a package of voluntary family planning and sexually transmitted infection (STI) information and services. BlueStar social franchisees—private providers accredited by MSM—are reimbursed for the cost of providing these services. We reviewed service statistics data from the first 18 months of the youth voucher program, from July 2013 to December 2014, as well as client demographic profile data from July 2015.

**Findings:** Between July 2013 and December 2014, 58,417 vouchers were distributed to young people by CHEs through a range of community mobilization efforts, of which 43,352 (74%) were redeemed for family planning and STI services. Most clients (78.5%) chose a long-acting reversible contraceptive (LARC), and just over half (51%) of young people benefited from STI counseling as part of their voucher service. Most (78%) services were provided in the Analamanga region (the capital and its surroundings), which was expected given the population density in this region and the high concentration of BlueStar franchisees. The client profile data snapshot from July 2015 revealed that 69% of voucher clients had never previously used a contraceptive method, and 96% of clients were aged 20 or younger, suggesting that the voucher program is successfully reaching the intended target group.

**Conclusion:**

MSM's youth voucher program has revealed a high demand for voluntary family planning services, especially among youth under 20 years old, and MSM has since integrated the youth voucher beyond the initial pilot locations. MSM's experience indicates that youth vouchers are a novel and effective means of increasing young people's access to voluntary family planning services in Madagascar, and this model could potentially be replicated or adapted in other contexts where young people are faced with barriers to accessing quality information and services.

## Abstract (precis)

Program accomplishments during the first 18 months:

- More than 58,000 free vouchers distributed to young people, of which 74% were redeemed.
- 79% chose long-acting reversible contraceptives (LARCs) and 51% received STI counseling.

Client profile data snapshot:

- 69% had never previously used contraception and 96% were 20 or younger.

## INTRODUCTION

Vouchers offer opportunities to reach specific groups of people with health services by removing financial barriers.^1^^,^^2^ The services provided with vouchers are typically highly specified—for example, family planning services—but generally allow the user to choose from a range of providers using a reimbursable token, often in paper form but sometimes digital.

The use of vouchers to support access to contraceptives can be traced back to Taiwan in the 1960s, where subsidized coupons were introduced to partially reimburse private-sector physicians for providing contraceptives under the national family planning program.^3^ Although the approach was slow to expand to other countries, there has been increased experimentation with voucher programs since 2000, with at least 30 programs in developing countries identified in a 2011 review of the use of vouchers in sexual and reproductive health (SRH) programs.^4^ Most voucher programs are used to engage the private sector, including NGOs, in subsidized service delivery to poorer women although a small number of programs have also experimented with using vouchers to increase access to public-sector facilities.

Voucher programs rarely target young people and/or adolescents exclusively. A recent systematic review of evaluations of family planning voucher programs identified only 2 studies of such programs: one in Vietnam focused on young people and another in Nicaragua focused on adolescents. No studies were identified on family planning voucher programs that focused on adolescents or young people in sub-Saharan Africa.^5^

Recent evaluations of family planning vouchers schemes have shown that vouchers have had a significant effect on voluntary contraceptive uptake in a range of settings.^6^^–^^8^ Vouchers appear to be more effective among poorer women with high demand to use contraception but who are less likely to access family planning due to higher prices.^6^

Marie Stopes International (MSI), an NGO providing family planning and SRH services in 37 countries, has introduced voucher schemes in several of its country programs to provide SRH services to the poorest and hard-to-reach populations, resulting in thousands of women accessing quality services. The success of these programs to date has been attributed to multiple factors such as targeted awareness-raising interventions, complemented by quality assurance measures and robust controls for voucher management. In many cases, the programs have engaged their private-sector social franchisee networks to deliver the services.^9^

### Madagascar Context

Madagascar is an island nation of 23 million inhabitants located off the east coast of Africa. Poverty levels are high; 92% of the population lives on less than US$2 per day.^10^ Almost two-thirds (64%) of the population is under 25 years of age,^11^ and there is a high rate of fertility among adolescents (163 births per 1,000 adolescents).^12^ Rates of maternal mortality are also high, with an estimated 478 deaths per 100,000 live births, and 17% of female adolescent deaths are due to maternal causes.^12^ The most recent Demographic and Health Survey (DHS) conducted in 2008–09 revealed that 17% of married women and 14% of unmarried, sexually active women aged 15–19 years were using a modern method of contraception, but 27% of married women of this age group expressed an unmet need for family planning.^13^ Long-acting reversible contraceptives (LARCs) were used by less than 0.5% of 15–19-year-olds, and are not readily available through the public sector, limiting contraceptive choices to short-acting methods. The same survey found high rates of teenage childbearing; 32% of women aged 15–19 years already had children or were currently pregnant, and this was significantly higher for the poorest quintile where 51% had started childbearing.^13^ Early marriage is common in Madagascar where 48% of girls are married before the age of 18.^14^

Marie Stopes Madagascar (MSM) has been operating in Madagascar since 1992 and is one of the largest non-state providers of voluntary family planning and SRH services. MSM delivers services to clients in all 22 regions of Madagascar through a network of 20 clinics; 200 MSI-trained midwives offering short-acting and voluntary LARC methods through mobile community visits; 22 mobile outreach teams; and 150 private and 140 public social franchisees. Social franchisees are existing, third-party private providers who are trained, accredited, and quality-monitored under MSI's social franchising brand, BlueStar (private sector) or CSBStar (public sector in Madagascar), to deliver a broad range of voluntary high-quality family planning services, including LARCs.

In 2012, MSM recognized that despite the high youth population of Madagascar and the high rates of early marriage, pregnancy, and unmet need for family planning, only 12% of BlueStar franchisee clients were aged 15–19 years. Cost is a key barrier for young people to access services, as documented by other MSI country programs,^15^ and the high levels of poverty in Madagascar indicated that cost should also be considered a key barrier for this age group.

Since 2011, MSM has been implementing a voucher program linked to the BlueStar franchise network whereby clients who are assessed as poor can purchase a voucher for Malagasy Ariary 200 (approximately US$0.06) and use the voucher to receive voluntary family planning counseling and services at a BlueStar facility for no additional fee.^16^ This program, aimed at reducing financial barriers for poor clients and expanding method choice, uses SMS reporting of voucher claims and mobile money payments to franchisees.^17^ In 2013, MSM began designing and developing a youth voucher program to improve youth's access to voluntary family planning services. This article describes the design of the youth voucher program and reviews service statistic data from the first 18 months of implementation.

Marie Stopes Madagascar (MSM) began a youth voucher program in 2013 to improve youth's access to voluntary family planning and STI services at BlueStar franchisees.

## METHODS

### Voucher Program Design and Development

To design the youth voucher program in Madagascar, MSM drew on its own voucher program management experience and on lessons from other MSI country programs that have used vouchers to reach young people. Lessons learned from a youth voucher pilot program in MSI's Zimbabwe country platform in 2013, funded by the United States Agency for International Development (USAID), confirmed that the provision of vouchers to young people for family planning services was welcome by recipients for overcoming the cost barrier of paying out of pocket for health services.^18^ Client and provider feedback on this pilot highlighted the need to expand the range of services available through the voucher to include services for sexually transmitted infections (STIs) and follow-up care for family planning services, e.g., removals or check-ups for voluntary LARC methods. As well as reducing cost barriers, voucher programs were considered a means to increase the range of voluntary contraceptive choices available, as well as the quality of services provided.

MSM was also aware that the overall use of mobile phones in Madagascar had increased dramatically, with an estimated 40% of the population owning a mobile phone,^19^ or 46 mobile phones per 100 people.^20^ A 2012 survey revealed that over half of MSM BlueStar clients owned mobile phones. MSM decided that mobile phones not only offered an opportunity to deliver vouchers electronically (i.e., eVouchers) to young people, but also that these could be seen as attractive and novel by MSM's target youth audience, increasing their appeal and thus distribution potential.

MSM adapted its existing voucher management software to incorporate the youth voucher component and trained community health educators (CHEs) and BlueStar franchisees on the new youth voucher. CHEs are contracted by MSM to provide quality counseling on comprehensive family planning and referral support at the community level to MSM BlueStar franchisees. They are based within or near the communities in which they work and distribute the poverty and youth vouchers to those eligible to receive them. To ensure that the needs of young people were appropriately addressed, MSM introduced youth-friendly training for the entire social franchise network of 150 BlueStar franchisees and for all CHEs. The training curriculum used by MSM, and delivered by MSM's Youth Medical Advisor, was adapted from the Ministry of Health's youth strategy and included a range of modules such as communication techniques, SRH of young people, and how to adapt service delivery to be youth-friendly.

MSM initially envisioned the youth voucher exclusively as an SMS-based mobile voucher. In parallel to commencing mobile voucher activities, MSM conducted a study to assess the acceptability of a mobile-based voucher program and to better understand how to reach young people who did not possess a mobile phone.^21^ Results revealed that young people were receptive to receiving family planning information through mobile phones, with a desire for more information on the range of contraceptive methods and where to access them. However, the study also highlighted the need for an alternative strategy as only 20% of respondents under the age of 20 years possessed a mobile phone. In addition, remote parts of Madagascar have limited access to phone network coverage. To maximize the program's reach, MSM adapted its voucher distribution system to include a paper voucher component.

The youth voucher program was initially envisioned as an SMS-based mobile program, but paper vouchers were added to ensure access for youth without mobile phones or living in remote areas without mobile phone coverage.

The voucher program commenced in late 2013 in 6 regions with 27 CHEs and 24 BlueStar franchisees, and expanded in scope over time, reaching 10 regions with 56 CHEs and 80 BlueStar franchisees by late 2014. More details of the voucher program methodology can be found in a program report.^22^

### Voucher Distribution

**FIGURE 1:**
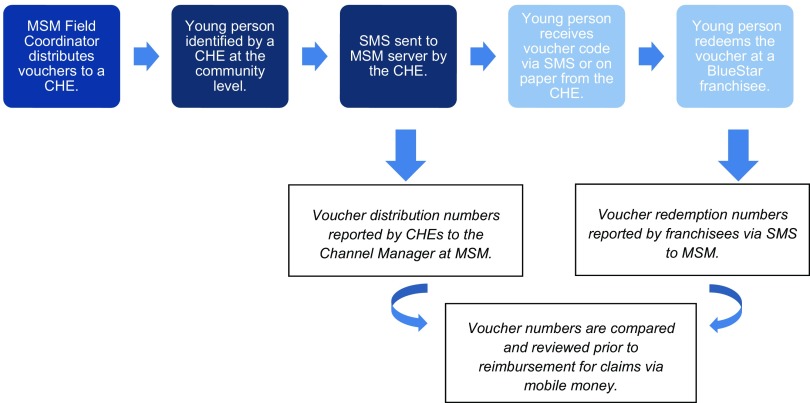
Distribution Process During the Initial Phase of the MSM Youth Voucher Program (July 2013–December 2014) Abbreviations: CHE, community health educator; MSM, Marie Stopes Madagascar.

During the initial phase of the voucher program, when an eligible young person expressed interest in receiving a service from a provider in the BlueStar network, the CHE initially provided him or her with a voucher in one of two ways (Figure 1):

1. If the young person had a mobile phone, the CHE would send an SMS to MSM to request an electronic voucher be sent to the young person's phone. This code formed the youth voucher, which the young person could then use to redeem for voluntary family planning and/or STI services at a participating BlueStar franchisee of his or her choice.
2. If the young person did not have a mobile phone or did not want an eVoucher sent, CHEs requested a voucher code (as described above) using their own mobile phone, and upon receipt, the CHEs wrote the code on a piece of paper for the young person to redeem for a free service.

### Voucher Package of Services

MSM designed a package of services covered under the voucher to meet the needs of young people. When a young person presents at a BlueStar facility with a voucher, the franchisee provides information and counseling about the voluntary family planning and STI services available at the clinic. The young person then decides if he or she wants a service, and if so, which one(s). These services are then provided at no cost to the client. The services available under the youth voucher program are:

- Family planning counseling
- Provision of short-acting contraceptive methods (oral contraceptive pill, 3-month injectable, emergency contraception, condoms)
- Provision of LARCs (3-year implant, 10-year IUD)
- STI screening, counseling, and referral for treatment
- Removal of LARC method

A young person is able to obtain a combination of services under one voucher, for example a contraceptive method of choice and STI counseling and screening.

Youth voucher program services included family planning counseling, provision of short-acting and long-acting methods, and STI counseling.

### Voucher Awareness Creation

MSM launched the youth voucher program with a comprehensive awareness-creation strategy to engage young people. All of MSM's CHEs—largely young women under the age of 25 years—distribute vouchers in their communities. Following a mapping exercise, CHEs identify eligible young people (male or female) aged 24 years or younger, with a focus on those under 20 years old, through their personal networks, concentrating mobilization efforts in areas where young people congregate, such as in and around schools and at youth associations and markets.

Community-based mobilization efforts by CHEs are complemented by other informational activities, such as concerts for young people where information on the vouchers is available, and community sensitization with influential community members such as parents, teachers, and community leaders. MSM also collaborates with organizations working with commercial sex workers in HIV prevention and support to refer young commercial sex workers for voluntary family planning and STI services using the voucher.

In addition, MSM introduced collaborative community mobilization efforts led by CHEs working together with BlueStar franchisees in their communities. The events take place either at or outside the franchisee facility. To increase service accessibility, some franchisees temporarily provide services outside their regular clinic, at sites within sensitized communities, following identification of sites with high potential youth demand for voucher services. If the site does not meet the necessary MSI clinical quality standards to provide services, the CHEs distribute the vouchers but young people are referred to the franchisee facility to redeem their voucher for a service(s).

This collaboration between the CHEs and franchisees to provide comprehensive information and voucher distribution coupled with same-place, same-time provision of services is appealing to young people, resulting in many young people redeeming their vouchers on the same day as the event.

### Quality Assurance and Support for BlueStar Providers

As part of its social franchising service delivery channel, MSM conducts ongoing quality assurance monitoring and support for BlueStar franchisees. Regular visits conducted by the medical team help ensure adherence to MSI's global quality standards and identify support requirements. BlueStar franchisees are included in annual MSM internal and external clinical audits to evaluate the quality of client care and service provision by individual providers. Client experience, one key indicator of service quality, is monitored annually through client exit interviews and mystery client visits. These measures, together with youth-friendly training received by each BlueStar franchisee participating in the voucher program, support providers to consistently deliver high-quality services that meet the needs of young clients.

Ongoing quality assurance monitoring coupled with training in provision of youth-friendly services have supported providers to consistently deliver high-quality services that meet the needs of young clients.

### Voucher Reimbursement to BlueStar Providers

**FIGURE 2:**
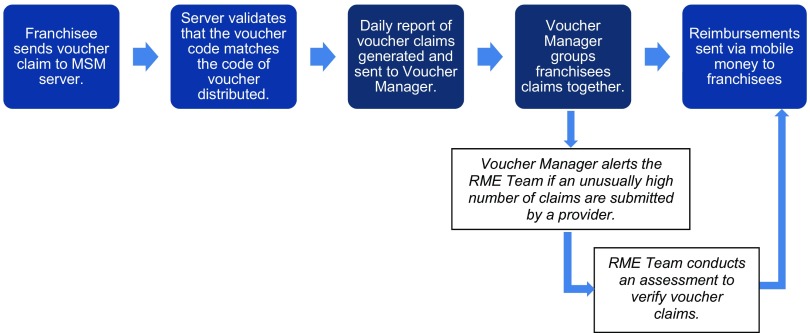
Marie Stopes Madagascar Youth Voucher Program Reimbursement and Verification Process Abbreviation: RME, Research, Monitoring, and Evaluation.

BlueStar providers deliver services free of charge to voucher-bearing young clients. Following service delivery, BlueStar providers submit claims for voucher services provided, and MSM reimburses providers for their costs. Following similar operating procedures for MSM's other, large-scale poverty voucher program, the franchisees send an SMS to MSM containing the required information, including the voucher code. MSM confirms that the SMS is from an approved mobile phone number and that the voucher code matches the database of voucher codes recorded as distributed by the CHE (Figure 2). This system allows MSM to reimburse providers promptly and identify operational issues through ongoing monitoring of real-time program data.

BlueStar franchisees submit reimbursement claims regularly, and payments are processed through online mobile money payment systems available in Madagascar, via the franchisees' mobile money network of choice. The amount reimbursed for each voucher varies depending on the type of service(s) provided (Table 1), as some services incur a higher commodity cost and take a longer timer to counsel on and provide, such as the provision of voluntary LARC methods. Set reimbursement rates for each service, or combination of services, are communicated to each participating franchisee prior to accreditation.

**TABLE 1.** Marie Stopes Madagascar Reimbursement Rates to BlueStar Franchisees for Youth Voucher Services

Service	Reimbursement Rate (MGA)	Reimbursement Rate (USD)^a^
IUD insertion	9,500	$4.00
Implant insertion	7,500	$3.20
Removal and reinsertion of an IUD	12,000	$5.10
Removal and reinsertion of an implant	10,000	$4.20
Removal of long-acting method for a short-acting method	2,000	$0.80
Short-acting method	2,000	$0.80
IUD plus STI counseling	12,500	$5.30
Implant plus STI counseling	12,500	$5.30
Short-acting method plus STI counseling	5,000	$2.10

Abbreviations: IUD, intrauterine device; MGA, Malagasy Ariary; STI, sexually transmitted infection; USD, U.S. dollars.

a Using the average exchange rate from Oanda.com for the period July 2013–December 2014 (US$1=2359 MGA).

### Monitoring and Verifying Claims

Monitoring visits by the MSM team provide the opportunity to cross-reference client data in daily registers with reimbursement claims made to MSM. The ongoing receipt of requests for voucher codes and claims for reimbursement permit real-time monitoring of the voucher program by MSM. Daily tracking of the number of voucher codes distributed by CHEs, the number of voucher codes redeemed, and the number of reimbursement claims enable MSM's Voucher Manager to follow up immediately in the event of data inconsistencies, including contacting franchisees if he/she experiences problems processing vouchers or submitting claims. Such real-time monitoring and provision of support would not be possible without using a system of unique codes that allows for continual submission of data.

An alert system was developed to identify instances of unusually high volume of claims submitted by a BlueStar franchisee. The Voucher Manager receives a daily report of voucher claims generated by providers. If the manager detects an unusually high number of claims submitted by a provider, the manager alerts the Research, Monitoring, and Evaluation (RME) team. In such instances, the RME team conducts a rapid voucher tracing assessment to check the validity of the claims by verifying client identities and reimbursed services (Figure 2). To ensure confidentiality, voucher clients provide their consent (or not) ahead of time to be contacted if their participation is required later for an assessment.

If services claimed by franchisees cannot be validated during the assessment process, MSM does not reimburse the franchisee. During the initial pilot phase of the youth voucher program, the assessment process was conducted on a monthly basis. We later shifted to conducting the process on a quarterly basis by selecting a random sample of franchisees for assessment. This mechanism highlights the importance of maintaining accurate client data records to ensure that claims can traced and validated.

### Data Collection and Analysis

In this article, we present service statistic data collected during the initial phase of the youth voucher program, from July 2013 to December 2014. Data include the number of vouchers distributed by CHEs and redeemed by youth, location of redemption, and types of services redeemed. MSM did not routinely collect demographic data on voucher clients during the initial pilot period of the program. Following the shift to 2 separate voucher distribution systems (eVouchers and paper vouchers), MSM commenced data collection to better understand the profile of the young people being reached. In this article, we present client profile data of paper voucher clients from July 2015. Client profile data were collected using smartphones, uploaded to a Microsoft Excel database, and numbers and percentages were extracted to generate a snapshot of the voucher clients' profile.

## RESULTS

During the initial phase of the youth voucher program, between July 2013 and December 2014, MSM distributed 58,417 vouchers, of which 43,352 were redeemed (74% redemption rate). As intended, most voucher redemptions (95%) occurred at BlueStar social franchisees, with only 4% redeemed at participating MSM outreach sites and 1% at MSM's Tulear region clinic.

**FIGURE 3:**
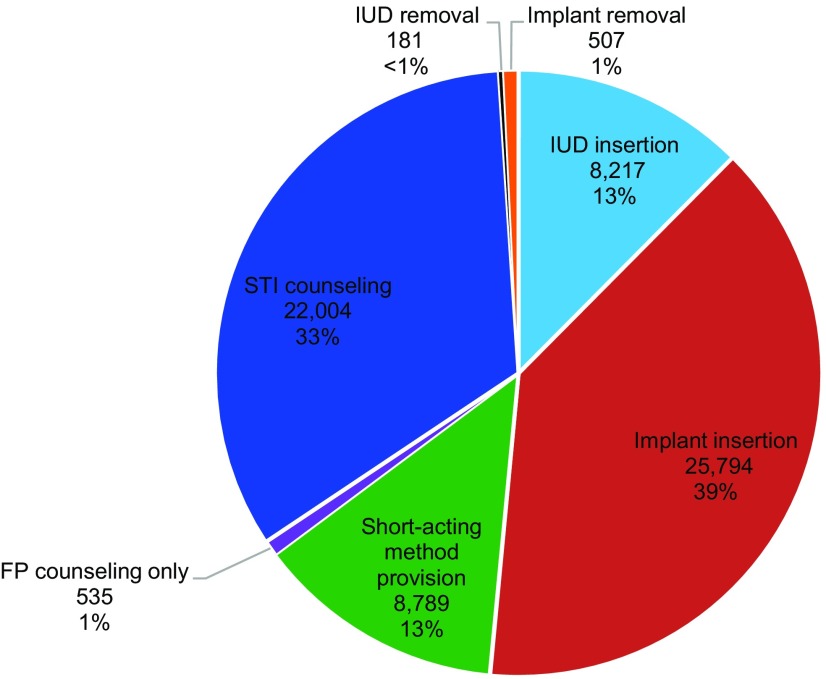
Services Redeemed by Young People With Vouchers, July 2013–December 2014 (N=66,027 Services) Abbreviations: FP, family planning; IUD, intrauterine device; STI, sexually transmitted infection.

**FIGURE 4:**
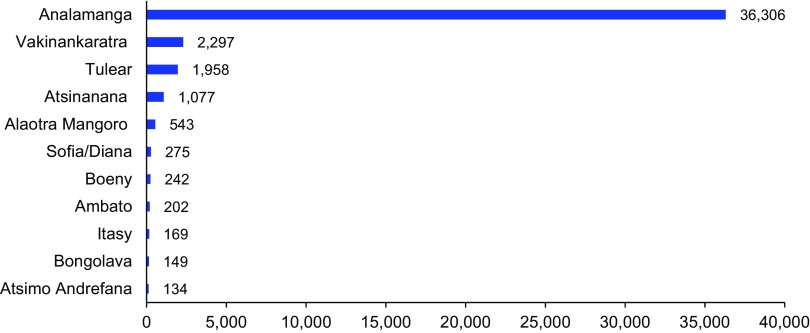
Number of Youth Vouchers Redeemed by Region, Marie Stopes Madagascar, July 2013–December 2014 (N=43,352 Vouchers Redeemed)

Voluntary LARCs were the most frequently chosen type of service (78.5%), with implants chosen 3 times as frequently as IUDs (Table 2). Some of these LARC services entailed removal of the IUD or implant, but in nearly all cases, the client had a new IUD or implant inserted. Short-acting methods were chosen by 20% of clients. About 1% of clients received family planning counseling only without provision of a method. Just over half of clients (51%) received STI counseling in addition to a contraceptive method. MSM reported that, in general, young people visited a service point seeking a voluntary family planning service. However, during history-taking, franchisee providers discovered that many young people were at risk of an STI (through the syndromic management approach), and subsequently many clients opted for STI counseling. BlueStar franchisees are reimbursed for the time taken to counsel a young person on STIs. As vouchers could be used to redeem one or a combination of services, Table 2 depicts these services separately (e.g., it separates “implant” from “implant plus STI counseling”). Figure 3 provides a more holistic picture of the volume of each type of service by grouping similar types of services. For example, all implant insertions are combined together (whether the client received only an implant insertion or an implant insertion plus STI counseling). The denominator in Figure 4 is thus the 66,027 total services provided. In the first year and a half of operation, the youth voucher program provided 25,794 implants, 8,217 IUDs, and 8,789 short-acting methods. The largest share (39%) of services was for insertion of an implant, followed by STI counseling (33%).

**TABLE 2.** Vouchers Redeemed by Type of Service, Marie Stopes Madagascar Youth Voucher Program, July 2013–December 2014 (N=43,352 Vouchers Redeemed)

Service	No. (%)
**Long-acting family planning services**	
IUD only	2,818 (6.5)
Implant only	11,608 (26.8)
IUD plus STI counseling	5,220 (12.0)
Implant plus STI counseling	13,682 (31.6)
Removal only of an IUD	2 (0.005)
Removal only of an implant	3 (0.007)
Removal and reinsertion of an IUD	179 (0.4)
Removal and reinsertion of an implant	504 (1.2)
**Total long-acting family planning services**	**34,016 (78.5)**
**Short-acting family planning services**	
Short-acting method only	5,564 (12.8)
Short-acting method plus STI counseling	3,102 (7.2)
Removal of long-acting method for a short-acting method	123 (0.3)
**Total short-acting family planning services**	**8,789 (20.3)**
**Other services**	
Family planning counseling only	535 (1.2)
Referrals	12 (0.03)
**Total other services**	**547 (1.3)**
**TOTAL**	**43,352 (100.0)**

Abbreviations: IUD, intrauterine device; STI, sexually transmitted infection.

### Results by Region

Youth vouchers were initially introduced in 4 regions; Analamanga, Mangoro, Tulear, and Vakinankaratra. By the end of the pilot period in December 2014, the vouchers had been extended to 11 of Madagascar's 22 regions where BlueStar franchisees and a significant youth population were concentrated. The highest proportion of voucher distribution and redemptions were in the region of Analamanga, where the capital city of Antananarivo is located, with 80% of all vouchers distributed and 78% of all vouchers redeemed in this region (Figure 4). This was expected, as Analamanga is the most densely populated region in Madagascar and has the highest concentration of BlueStar franchisees.

### Results by Demographic Characteristics

Table 3 provides a snapshot of characteristics of the 1,425 young people who used paper vouchers to receive a service in July 2015. Most (91%) of these youth voucher users were women, and the large majority (96%) were 20 years old or younger, suggesting that the voucher program is successfully reaching the intended target group. While the majority (53%) did not have any children, a significant proportion (46%) had one or more children. Over a third (39%) were married or cohabiting with a partner. Of the 1,425 young people, 69% had never previously used a contraceptive method.

**TABLE 3.** Paper Voucher Client Profile, Marie Stopes Madagascar, July 2015 (N=1,425)

Client Characteristic	No. (%)
Sex	
Female	1303 (91)
Male	122 (9)
Age, years	
<15	75 (5)
15–18	786 (55)
19–20	514 (36)
21–24	50 (4)
No. of living children	
0	757 (53)
1	502 (35)
2	148 (10)
3	14 (1)
5 or more	4 (0.3)
Marital status	
Married or cohabiting	553 (39)
Single or living alone	872 (61)
First-time family planning user	
Yes	987 (69)
No	438 (31)

## DISCUSSION

The MSM voucher program enabled a significant number of young people in Madagascar to access a range of voluntary family planning and STI information and services, while increasing the capacity of BlueStar franchisees to provide high-quality services to this key group of people. The voucher program expanded young people's choice of contraceptive methods to include voluntary LARCs, which the public sector to date has not been able to fulfill. (See the Box for a story of a youth voucher client.)

The MSM voucher program expanded young people's choice of contraceptive methods to include voluntary LARCs.

BOX **Marie Stopes Madagascar Youth Voucher Client Story**

One of Marie Stopes Madagascar's (MSM's) youth voucher beneficiaries is a 17-year-old who has a 2-month-old baby. She has 8 siblings and left primary school when she was 9 years old because her mother could not afford to keep sending her. She is unemployed but her fiancé works at the bus station, earning between US$1.70 and $6.00 a day. Following a visit to Dr. Florence's BlueStar clinic for her baby's vaccinations, she met one of MSM's community health educators and learned about the voucher program for young people. Having never previously used a contraceptive method, she chose to redeem her voucher for an IUD. Without the voucher program, her only option to access family planning services would have been at a public health center, limiting her to only short-acting methods since long-acting methods are not widely available in the public sector.

More than 43,000 young people accessed services in the voucher program's first 18 months of operation. These impressive results demonstrate that need exists among young people for high-quality family planning information and a broad range of voluntary services. The success of the voucher program is likely due to a combination of factors: on the demand side, the provision of community-based information, education, and communication activities to young people and other community members and the removal of service fees; and on the supply side, the availability of a broad range of contraceptive methods and a quality provider of the client's choice with the willingness and capacity to serve young people. These complementary components of the voucher program led to a significant redemption of the vouchers, and could be a demonstration of young people feeling empowered to make informed choices about their SRH.^23^ The voucher program also highlighted the high demand for voluntary LARCs among young people if they are made available to them; over 4 times as many voluntary LARCs were chosen by young people compared with short-acting methods when they were provided with a broad choice of methods.

The program demonstrated the ability of vouchers to leverage private provider networks to provide quality services to a large number of young people, and MSM has been impressed by the willingness, and increasing confidence, of private-sector providers to provide voluntary family planning and STI information and services to this group.

Lessons have been learned along the way, especially related to the absence of mobile phones among young people in poorer communities. The voucher distribution system was quickly adapted, and later refined, to provide a paper-based voucher to complement the eVoucher, enabling access for young people without mobile phones while ensuring robust voucher tracking and follow-up. CHEs now distribute only the paper voucher at the community level. Meanwhile, MSM's Call Centre—a free hotline where callers receive family planning information and referrals to service providers—provides an opportunity to reach young people with mobile phones who may not have been reached via community-based CHE activities to date. This two-pronged approach will undoubtedly increase access to voluntary family planning and STI services for young people in the future. The voucher system will continue to evolve over time, as MSM fine-tunes operations and responds to client feedback. Systems will be continuously reviewed to ensure that the distribution, redemption, and claims processes are robust and efficient. Quarterly assessments of a random selection of social franchisees is an example of how one of MSM's monitoring activities has evolved to enhance data accuracy and identify any necessary support requirements.

## CONCLUSION

MSM's experience indicates that youth vouchers can be a novel and effective means of increasing access to voluntary family planning services for young people, and that the private sector can be an appropriate and acceptable delivery channel to deliver a broad range of voluntary contraceptive methods for young people. MSM's approach of using vouchers to increase awareness and support to improve supply has the potential to be replicated (with adaptation to context) and taken to scale in other countries where young people face barriers in accessing voluntary, quality information and services, thus increasing family planning access and choice for the growing youth cohort in developing countries.

## Notes

**Peer Reviewed**

**First Published Online:** 2017 Feb 23
